# 4,4-Bis(1*H*-pyrrol-2-yl)penta­nol

**DOI:** 10.1107/S1600536809054269

**Published:** 2010-01-16

**Authors:** Guillaume Journot, Reinhard Neier, Helen Stoeckli-Evans

**Affiliations:** a Institute of Chemistry, University of Neuchâtel, rue Emile-Argand 11, 2009 Neuchâtel, Switzerland; b Institute of Physics, University of Neuchâtel, rue Emile-Argand 11, 2009 Neuchâtel, Switzerland

## Abstract

The title achiral compound, C_13_H_18_N_2_O, crystallized in the chiral monoclinic space group *P*2_1_. The pyrrole rings are inclined to one another by 62.30 (11)°, and the propanol chain is in an extended conformation. In the crystal, the two pyrrole NH groups are involved in inter­molecular N—H⋯O hydrogen bonds, leading to the formation of a helical arrangement propagating along the *b* axis. An inter­esting feature of the crystal structure is the absence of any conventional hydrogen bonds involving the hydr­oxy H atom. There is, however, a weak inter­molecular O—H⋯π inter­action involving one of the pyrrole rings.

## Related literature

For substituted calix[4]pyrroles, see: Gale *et al.* (1998 ▶); Sessler & Davis (2001 ▶); Sessler *et al.* (2003 ▶). For the crystal structures of similar compounds, see: Warriner *et al.* (2003 ▶); Maeda *et al.* (2007 ▶); Sobral *et al.* (2003 ▶). For details of hydrogen-bonding graph-set analysis, see: Bernstein *et al.* (1995 ▶). For a description of the Cambridge Structural Database, see: Allen (2002 ▶).
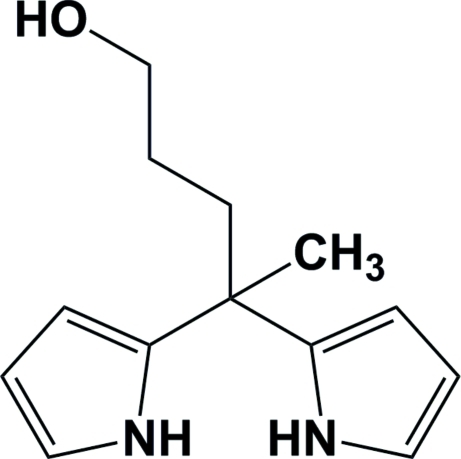


## Experimental

### 

#### Crystal data

C_13_H_18_N_2_O
*M* *_r_* = 218.29
Monoclinic, 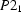

*a* = 8.4721 (15) Å
*b* = 8.2111 (9) Å
*c* = 8.7120 (15) Å
β = 101.530 (14)°
*V* = 593.82 (16) Å^3^
*Z* = 2
Mo *K*α radiation
μ = 0.08 mm^−1^
*T* = 173 K
0.45 × 0.45 × 0.40 mm

#### Data collection

Stoe IPDS-2 diffractometer
6119 measured reflections
1701 independent reflections
1518 reflections with *I* > 2σ(*I*)
*R*_int_ = 0.032

#### Refinement

*R*[*F* ^2^ > 2σ(*F* ^2^)] = 0.030
*wR*(*F* ^2^) = 0.077
*S* = 0.97
1701 reflections
159 parameters
1 restraint
H atoms treated by a mixture of independent and constrained refinement
Δρ_max_ = 0.19 e Å^−3^
Δρ_min_ = −0.16 e Å^−3^

### 

Data collection: *X-AREA* (Stoe & Cie, 2009 ▶); cell refinement: *X-AREA*; data reduction: *X-RED32* (Stoe & Cie, 2009 ▶); program(s) used to solve structure: *SHELXS97* (Sheldrick, 2008 ▶); program(s) used to refine structure: *SHELXL97* (Sheldrick, 2008 ▶); molecular graphics: *ORTEP-3* (Farrugia, 1997 ▶) and *Mercury* (Macrae *et al.*, 2006 ▶); software used to prepare material for publication: *SHELXL97* and *PLATON* (Spek, 2009 ▶).

## Supplementary Material

Crystal structure: contains datablocks I, global. DOI: 10.1107/S1600536809054269/is2505sup1.cif

Structure factors: contains datablocks I. DOI: 10.1107/S1600536809054269/is2505Isup2.hkl

Additional supplementary materials:  crystallographic information; 3D view; checkCIF report

## Figures and Tables

**Table 1:** Hydrogen-bond geometry (Å, °)

*D*—H⋯*A*	*D*—H	H⋯*A*	*D*⋯*A*	*D*—H⋯*A*
N1—H1*N*⋯O1^i^	0.88 (2)	2.05 (2)	2.9238 (18)	174.3 (19)
N2—H2*N*⋯O1^ii^	0.90 (2)	2.06 (2)	2.9529 (18)	171.5 (19)
O1—H1*O*⋯*Cg*1^iii^	0.87 (3)	2.53	3.20	135
O1—H1*O*⋯*Cg*2^iii^	0.87 (3)	2.64	3.10	114

Symmetry codes: (i) 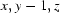; (ii) 
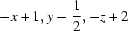
; (iii) 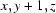. *Cg*1 and *Cg*2 are the centroids of the C7=C8 bond and the N2/C5–C8 pyrrole ring, respectively.

**Fig. 1.**
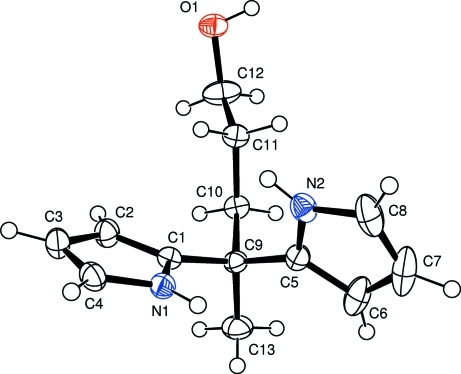
A view of the molecular structure of the title compound, with the displacement ellipsoids drawn at the 50% probability level.

**Fig. 2.**
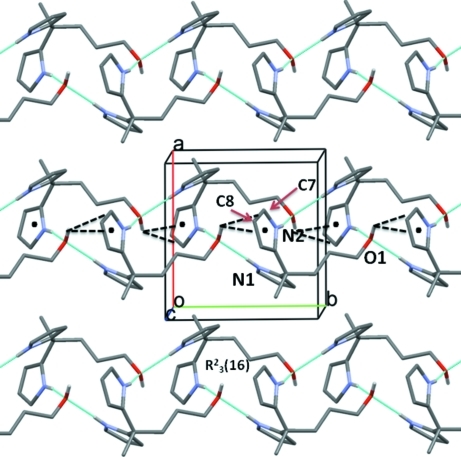
A view, along the c axis, of the crystal packing of the title compound. The N—H···O hydrogen bonds are shown as dotted cyan lines and the O—H···π interactions as dotted black lines [for clarity these interactions are shown for only one of the helices; see Table 1 for details].

## supplementary crystallographic information

### Comment

The title compound (systematic name: 4,4-di(1*H*-pyrrol-2-yl)pentan-1-ol) was prepared as a building block for the formation of substituted calix[4]pyrroles. The latter have been shown to form extremely interesting host–guest complexes with various anions (Gale *et al.*, 1998; Sessler and Davis, 2001; Sessler *et al.*, 2003).

The structure of the title compound is shown in Fig. 1, and the geometrical parameters are given in the Supplementary Information and the archived CIF. This achiral compound crystallized in the chiral monoclinic space group *P*2_1_. The bond lengths and angles are similar to those observed in 5 similar 1,1-bis(2-pyrrolyl)ethane compounds in the Cambridge Crystal Structure Database (CSD, V5.30, last update Sep. 2009; Allen *et al.*, 2002). These include the (3,4,5-tribromo-2-pyrrolyl) derivative (Warriner *et al.*, 2003; AJARIM), the *o*-, *m*- and *p*-pyridyl derivaties (Maeda *et al.*, 2007; CIGKIN, CIGKEJ, CIGKAF, respectively) and the phenyl derivative (Sobral *et al.*, 2003; JADHUS), all of which crystallized as racemates.

In the title compound the pyrrole ring mean-planes are inclined to one another by 62.30 (11)°, and the propanol chain is in the extended conformation. In the 5 compounds located in the CSD this angle varies between 68.5 to 89.6 °.

In the crystal the molecules are linked by conventional N—H···N intermolecular hydrogen bonds leading to the formation of helical chains propagating along the *b* axis (Fig. 2 and Table 1). The basic unitary hydrogen bonding graph set can be described by an *R*^2^_3_(16) ring, while the basic binary graph set is a C(8) chain. This gives an extended notation of C(8)[*R*^2^_3_(16)] (Bernstein *et al.*, 1995). A fuller hydrogen bonding graph set analysis can be obtained using the program Mercury (Macrea *et al.*, 2006).

An O—H···π interaction is also observed in the crystal structure (Fig. 2 and Table 1). It can be considered either to involve the C7═C8 bond (centroid = *Cg*1) with an O—H···π angle of *ca* 135°, or a weaker interaction involving the pyrrole ring (N2/C5—C8; centroid = *Cg*2), with an O—H···π angle of only *ca* 114° [these data were obtained using the program Mercury (Macrae *et al.*, 2006)].

### Experimental

A mixture of 3-acetylpropanol (10 ml, 98.6 mmol) and pyrrole (50 ml, 0.720 mol) were stirred for 5 min and then trifluoro acetic acid [TFA] (0.74 ml, 9.6 mmol, 0.097 equiv.) was added. The whole mixture was stirred foran additional 5 min and then quenched with aqueous NaOH (0.1 N, 30 ml). The mixture was extracted with CH_2_Cl_2_ (50 ml × 2) and the organic layer dried (Na_2_SO_4_). The solvent was removed in *vacuo* and the remaining oil crystallized with dichloromethane (20 ml). The colourless block-like crystals obtained were washed with 2-propanol [m.p. 372 K; Yield 14.1 g (65.3%)]. ^1^H NMR (CDCl_3_) δ 7.85 (bs, 2H, N—H), 6.63–6.61 (ddd, J = 2.7 Hz,2.7 Hz, 1.6 Hz, 2H, pyrrolic-H_1–8_), 6.15–6.13 (ddd, J = 3.3 Hz, 2.7 Hz, 1.6 Hz, 2H, pyrrolic-H_2–7_), 6.10–6.08 (ddd, J = 3.3 Hz, 1.6 Hz,1.6 Hz, 2H, pyrrolic-H_3–6_), 3.61–3.57 (td, J = 6 Hz, 5 Hz, 2H, –O—CH2_12_), 2.07–2.03 (m, 2H, –CH2_10_–), 1.59 (s, 3H, –CH3_13_), 1.51–1.43 (m, 2H,-CH2_11_–), 1.24–1.20 (t, J = 5 Hz, 1H, –OH); ^13^C NMR (CDCl_3_)δ 137.97 (C_4–5_), 117.15 (C_1–5_), 107.92 (C_2–7_),104.77 (C_3–6_), 63.26 (C_12_), 39.04 (C_9_), 37.35 (C_10_),28.01 (C_11_), 26.62 (C_13_). MS calcd. for C_13_H_18_N_2_O 218.14, found 217.13 (M—H^+^).

### Refinement

In the final cycles of refinement, in the absence of significant anomalous scattering effects, 1239 Friedel pairs were merged and Δf " set to zero. The OH and NH H-atoms, located in a difference electron-density map, were freely refined: O—H = 0.83 (3) Å; N—H = 0.88 (2) - 0.90 (2) Å. The C-bound H-atoms were included in calculated positions and treated as riding atoms: C—H = 0.95, 0.99 and 0.98 Å for CH, CH_2_ and CH_3_ H-atoms, respectively, with *U*_iso_(H) = k × *U*_eq_(C), where k = 1.2 for CH and CH_2_ H-atoms, and 1.5 for CH_3_ H-atoms.

### Crystal data

**Table:**

C_13_H_18_N_2_O	*F*(000) = 236
*M**_r_* = 218.29	*D*_x_ = 1.221 Mg m^−^^3^
Monoclinic, *P*2_1_	Mo *K*α radiation, λ = 0.71073 Å
Hall symbol: P 2yb	Cell parameters from 5671 reflections
*a* = 8.4721 (15) Å	θ = 2.4–29.6°
*b* = 8.2111 (9) Å	µ = 0.08 mm^−^^1^
*c* = 8.7120 (15) Å	*T* = 173 K
β = 101.530 (14)°	Block, colourless
*V* = 593.82 (16) Å^3^	0.45 × 0.45 × 0.40 mm
*Z* = 2	

### Data collection

**Table:**

Stoe IPDS-2 diffractometer	1518 reflections with *I* > 2σ(*I*)
Radiation source: fine-focus sealed tube	*R*_int_ = 0.032
graphite	θ_max_ = 29.2°, θ_min_ = 2.4°
φ ans ω scans	*h* = −10→11
6119 measured reflections	*k* = −11→11
1701 independent reflections	*l* = −11→11

### Refinement

**Table:**

Refinement on *F*^2^	Secondary atom site location: difference Fourier map
Least-squares matrix: full	Hydrogen site location: inferred from neighbouring sites
*R*[*F*^2^ > 2σ(*F*^2^)] = 0.030	H atoms treated by a mixture of independent and constrained refinement
*wR*(*F*^2^) = 0.077	*w* = 1/[σ^2^(*F*_o_^2^) + (0.0571*P*)^2^] where *P* = (*F*_o_^2^ + 2*F*_c_^2^)/3
*S* = 0.97	(Δ/σ)_max_ < 0.001
1701 reflections	Δρ_max_ = 0.19 e Å^−^^3^
159 parameters	Δρ_min_ = −0.16 e Å^−^^3^
1 restraint	Extinction correction: *SHELXL97* (Sheldrick, 2008), Fc^*^=kFc[1+0.001xFc^2^λ^3^/sin(2θ)]^-1/4^
Primary atom site location: structure-invariant direct methods	Extinction coefficient: 0.108 (11)

### Special details

**Table:**

Geometry. Bond distances, angles *etc*. have been calculated using the rounded fractional coordinates. All su's are estimated from the variances of the (full) variance-covariance matrix. The cell e.s.d.'s are taken into account in the estimation of distances, angles and torsion angles
Refinement. In the final cycles of refinement, in the absence of significant anomalous scattering effects, 1239 Friedel pairs were merged and Δf " set to zero. The OH and NH hydrogen atoms were located in difference electron-density maps and were freely refined. The C-bound H-atoms were included in calculated positions and treated as riding.

### Fractional atomic coordinates and isotropic or equivalent isotropic displacement parameters (Å^2^)

**Table:**

	*x*	*y*	*z*	*U*_iso_*/*U*_eq_	
O1	0.41371 (13)	1.28786 (14)	0.88057 (13)	0.0300 (3)	
N1	0.24737 (14)	0.55996 (17)	0.99568 (13)	0.0244 (3)	
N2	0.48148 (15)	0.66563 (17)	0.79634 (16)	0.0281 (3)	
C1	0.18516 (15)	0.69959 (18)	0.92203 (15)	0.0220 (3)	
C2	0.12554 (18)	0.7921 (2)	1.02911 (17)	0.0293 (4)	
C3	0.15673 (18)	0.7047 (2)	1.17356 (17)	0.0318 (4)	
C4	0.23207 (17)	0.5631 (2)	1.14900 (16)	0.0286 (4)	
C5	0.33030 (17)	0.64998 (19)	0.70462 (15)	0.0248 (4)	
C6	0.3443 (2)	0.5545 (3)	0.57848 (18)	0.0409 (5)	
C7	0.5085 (3)	0.5108 (3)	0.5947 (2)	0.0518 (7)	
C8	0.5900 (2)	0.5804 (3)	0.7302 (2)	0.0419 (6)	
C9	0.18427 (16)	0.72822 (17)	0.74921 (15)	0.0221 (3)	
C10	0.17922 (17)	0.91270 (19)	0.71418 (16)	0.0244 (4)	
C11	0.32247 (16)	1.01255 (19)	0.79998 (16)	0.0248 (4)	
C12	0.2876 (2)	1.1921 (2)	0.7865 (2)	0.0398 (5)	
C13	0.03085 (19)	0.6525 (2)	0.65082 (18)	0.0349 (4)	
H1N	0.297 (2)	0.482 (3)	0.955 (2)	0.037 (5)*	
H1O	0.485 (4)	1.307 (4)	0.823 (3)	0.073 (9)*	
H2	0.07350	0.89480	1.01050	0.0350*	
H2N	0.504 (2)	0.709 (3)	0.893 (2)	0.034 (5)*	
H3	0.12990	0.73890	1.26930	0.0380*	
H4	0.26760	0.48110	1.22500	0.0340*	
H6	0.25910	0.52330	0.49530	0.0490*	
H7	0.55360	0.44540	0.52450	0.0620*	
H8	0.70230	0.57120	0.77130	0.0500*	
H10A	0.17120	0.92760	0.60010	0.0290*	
H10B	0.07980	0.95800	0.74090	0.0290*	
H11A	0.34710	0.98110	0.91190	0.0300*	
H11B	0.41830	0.98810	0.75510	0.0300*	
H12A	0.27480	1.22540	0.67550	0.0480*	
H12B	0.18470	1.21430	0.82010	0.0480*	
H13A	−0.06410	0.70130	0.68090	0.0520*	
H13B	0.03100	0.53480	0.66940	0.0520*	
H13C	0.02780	0.67330	0.53950	0.0520*	

### Atomic displacement parameters (Å^2^)

**Table:**

	*U*^11^	*U*^22^	*U*^33^	*U*^12^	*U*^13^	*U*^23^
O1	0.0322 (5)	0.0208 (6)	0.0362 (5)	−0.0021 (4)	0.0048 (4)	−0.0009 (4)
N1	0.0277 (6)	0.0221 (6)	0.0244 (5)	−0.0005 (5)	0.0073 (4)	0.0010 (5)
N2	0.0256 (5)	0.0262 (7)	0.0345 (6)	0.0036 (5)	0.0106 (5)	0.0033 (5)
C1	0.0204 (5)	0.0215 (7)	0.0239 (6)	−0.0020 (5)	0.0043 (4)	0.0001 (5)
C2	0.0302 (7)	0.0289 (8)	0.0307 (7)	0.0036 (6)	0.0108 (5)	−0.0001 (6)
C3	0.0331 (7)	0.0384 (9)	0.0262 (6)	−0.0018 (7)	0.0118 (5)	−0.0011 (6)
C4	0.0299 (7)	0.0320 (8)	0.0249 (6)	−0.0035 (6)	0.0078 (5)	0.0046 (6)
C5	0.0321 (7)	0.0208 (7)	0.0224 (6)	0.0019 (6)	0.0073 (5)	0.0027 (5)
C6	0.0604 (11)	0.0407 (10)	0.0228 (6)	0.0155 (9)	0.0113 (6)	0.0012 (6)
C7	0.0729 (13)	0.0529 (13)	0.0382 (9)	0.0297 (11)	0.0318 (9)	0.0085 (9)
C8	0.0396 (8)	0.0425 (11)	0.0505 (10)	0.0156 (8)	0.0257 (7)	0.0153 (8)
C9	0.0231 (6)	0.0217 (7)	0.0206 (5)	−0.0018 (5)	0.0025 (4)	−0.0003 (5)
C10	0.0245 (6)	0.0230 (7)	0.0243 (6)	0.0015 (5)	0.0016 (5)	0.0018 (5)
C11	0.0241 (6)	0.0200 (7)	0.0290 (6)	0.0013 (5)	0.0025 (5)	0.0026 (5)
C12	0.0364 (8)	0.0217 (9)	0.0537 (10)	0.0023 (7)	−0.0094 (7)	−0.0004 (7)
C13	0.0336 (7)	0.0372 (9)	0.0303 (7)	−0.0103 (7)	−0.0021 (5)	−0.0021 (7)

### Geometric parameters (Å, °)

**Table:**

O1—C12	1.443 (2)	C10—C11	1.530 (2)
O1—H1O	0.87 (3)	C11—C12	1.504 (2)
N1—C1	1.367 (2)	C2—H2	0.9500
N1—C4	1.3678 (18)	C3—H3	0.9500
N2—C8	1.371 (2)	C4—H4	0.9500
N2—C5	1.3735 (19)	C6—H6	0.9500
N1—H1N	0.88 (2)	C7—H7	0.9500
N2—H2N	0.899 (19)	C8—H8	0.9500
C1—C9	1.5224 (18)	C10—H10A	0.9900
C1—C2	1.375 (2)	C10—H10B	0.9900
C2—C3	1.427 (2)	C11—H11A	0.9900
C3—C4	1.364 (2)	C11—H11B	0.9900
C5—C6	1.374 (2)	C12—H12A	0.9900
C5—C9	1.512 (2)	C12—H12B	0.9900
C6—C7	1.416 (3)	C13—H13A	0.9800
C7—C8	1.368 (3)	C13—H13B	0.9800
C9—C10	1.544 (2)	C13—H13C	0.9800
C9—C13	1.538 (2)		
			
C12—O1—H1O	106.9 (19)	C4—C3—H3	126.00
C1—N1—C4	109.86 (13)	N1—C4—H4	126.00
C5—N2—C8	109.48 (13)	C3—C4—H4	126.00
C4—N1—H1N	123.7 (13)	C5—C6—H6	126.00
C1—N1—H1N	126.3 (13)	C7—C6—H6	126.00
C5—N2—H2N	125.5 (11)	C6—C7—H7	126.00
C8—N2—H2N	124.2 (12)	C8—C7—H7	126.00
N1—C1—C2	107.72 (12)	N2—C8—H8	126.00
N1—C1—C9	121.26 (12)	C7—C8—H8	126.00
C2—C1—C9	130.98 (13)	C9—C10—H10A	108.00
C1—C2—C3	106.99 (14)	C9—C10—H10B	108.00
C2—C3—C4	107.48 (13)	C11—C10—H10A	108.00
N1—C4—C3	107.94 (13)	C11—C10—H10B	108.00
N2—C5—C6	107.40 (14)	H10A—C10—H10B	107.00
N2—C5—C9	121.77 (12)	C10—C11—H11A	109.00
C6—C5—C9	130.82 (13)	C10—C11—H11B	109.00
C5—C6—C7	107.75 (15)	C12—C11—H11A	109.00
C6—C7—C8	107.25 (18)	C12—C11—H11B	109.00
N2—C8—C7	108.11 (17)	H11A—C11—H11B	108.00
C1—C9—C10	109.96 (11)	O1—C12—H12A	109.00
C1—C9—C5	110.24 (11)	O1—C12—H12B	109.00
C5—C9—C10	110.97 (12)	C11—C12—H12A	109.00
C5—C9—C13	109.20 (12)	C11—C12—H12B	109.00
C1—C9—C13	108.99 (11)	H12A—C12—H12B	108.00
C10—C9—C13	107.43 (11)	C9—C13—H13A	109.00
C9—C10—C11	116.18 (12)	C9—C13—H13B	109.00
C10—C11—C12	111.28 (12)	C9—C13—H13C	109.00
O1—C12—C11	112.24 (13)	H13A—C13—H13B	109.00
C1—C2—H2	127.00	H13A—C13—H13C	109.00
C3—C2—H2	126.00	H13B—C13—H13C	110.00
C2—C3—H3	126.00		
			
C4—N1—C1—C2	−1.41 (16)	N2—C5—C6—C7	0.2 (2)
C4—N1—C1—C9	−179.27 (12)	C9—C5—C6—C7	−178.96 (17)
C1—N1—C4—C3	1.08 (17)	N2—C5—C9—C1	−45.13 (18)
C8—N2—C5—C6	−0.4 (2)	N2—C5—C9—C10	76.94 (16)
C8—N2—C5—C9	178.81 (15)	N2—C5—C9—C13	−164.83 (14)
C5—N2—C8—C7	0.5 (2)	C6—C5—C9—C1	133.92 (18)
N1—C1—C2—C3	1.17 (17)	C6—C5—C9—C10	−104.0 (2)
C9—C1—C2—C3	178.74 (14)	C6—C5—C9—C13	14.2 (2)
N1—C1—C9—C5	−32.86 (18)	C5—C6—C7—C8	0.1 (2)
N1—C1—C9—C10	−155.52 (13)	C6—C7—C8—N2	−0.4 (3)
N1—C1—C9—C13	86.97 (15)	C1—C9—C10—C11	61.87 (15)
C2—C1—C9—C5	149.85 (15)	C5—C9—C10—C11	−60.36 (15)
C2—C1—C9—C10	27.2 (2)	C13—C9—C10—C11	−179.65 (12)
C2—C1—C9—C13	−90.32 (18)	C9—C10—C11—C12	−166.90 (12)
C1—C2—C3—C4	−0.53 (18)	C10—C11—C12—O1	173.65 (12)
C2—C3—C4—N1	−0.33 (17)		

### Hydrogen-bond geometry (Å, °)

**Table:**

Cg1 and Cg2 are the centroids of the C7═C8 bond and the N2/C5–C8 pyrrole ring, respectively.

**Table:**

*D*—H···*A*	*D*—H	H···*A*	*D*···*A*	*D*—H···*A*
N1—H1N···O1^i^	0.88 (2)	2.05 (2)	2.9238 (18)	174.3 (19)
N2—H2N···O1^ii^	0.90 (2)	2.06 (2)	2.9529 (18)	171.5 (19)
O1—H1O···Cg1^iii^	0.87 (3)	2.53	3.20	135
O1—H1O···Cg2^iii^	0.87 (3)	2.64	3.10	114

Symmetry codes: (i) *x*, *y*−1, *z*; (ii) −*x*+1, *y*−1/2, −*z*+2; (iii) *x*, *y*+1, *z*.
